# Burning Down the House: Cellular Actions during Pyroptosis

**DOI:** 10.1371/journal.ppat.1003793

**Published:** 2013-12-19

**Authors:** Christopher N. LaRock, Brad T. Cookson

**Affiliations:** 1 Department of Microbiology, University of Washington, Seattle, Washington, United States of America; 2 Department of Laboratory Medicine, University of Washington, Seattle, Washington, United States of America; University of North Carolina at Chapel Hill School of Medicine, United States of America

## Introduction

**Figure 1:**
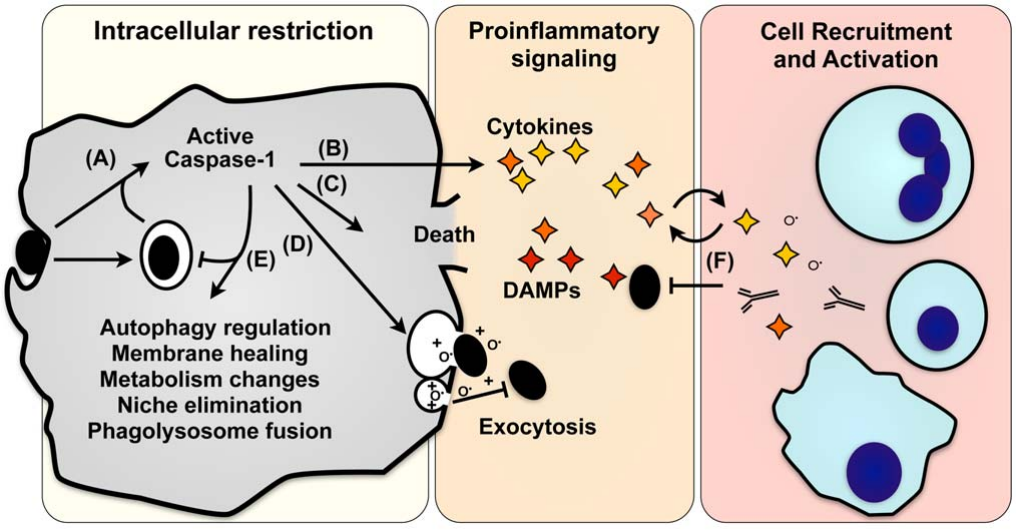
Effector mechanisms of pyroptosis. (**A**) Caspase-1 activation in response to microbial infection (black ovals) initiates numerous pathways that promote death or recovery of the cell, directly combat pathogen infection, or signal to other cells. (**B**) The proinflammatory cytokines IL-1β and IL-18 are cleaved and secreted, and HMGB1, IL-1α, FGF2, and numerous damage-associated molecular patterns are also released. (**C**) Caspase-1 can also initiate programmed cell death, eliminating a niche for intracellular pathogens while releasing both pathogen and proinflammatory signals. (**D**) Intracellular pathogens and antimicrobial factors that kill extracellular bacteria can be released by lysosomal exocytosis, also promoting adaptive immune responses through cross-priming. (**E**) Caspase-1 promotes cellular integrity by removing microbes or damaged organelles by stimulating autophagy, enhanced lysosome activity, induction of lipid metabolism, and exocytosis of damaged or infected organelles. (**F**) Proinflammatory signals released by lysis, exocytosis, and other secretory pathways recruit and activate immune cells (blue; clockwise from top: neutrophils, lymphocytes, macrophages/dendritic cells). The specific responses of a cell vary depending on the kinetics and magnitude of caspase-1 stimulation, the activating stimulus, and cell type.

Under threat of pathogen invasion, timely initiation of inflammation is a critical first step in generating a protective immune response. One major obstacle for the immune system is discriminating pathogenic microbes from non-pathogenic microbiota. Some members of the nucleotide-binding oligomerization domain-like receptor (NLR) family of pattern recognition receptors accomplish this distinction based on localization—typically, only pathogens deliver NLR ligands into the cytosol, where these receptors are localized. Ultimately, these NLRs initiate assembly of an inflammasome complex that activates the proteases caspase-1 and caspase-11. These caspases were originally identified for their role in IL-1β processing and release but are now known to direct additional important cellular processes during infection, inflammatory disorders, and response to injury. In this brief review, we enumerate these emerging pathways (Figure 1) and highlight their roles in disease.

## IL-1β and IL-18

The proinflammatory cytokines IL-1β and IL-18 were the earliest studied caspase-1 substrates. IL-1β directs diverse processes, including extravasation, cell proliferation and differentiation, cytokine secretion, angiogenesis, wound healing, and pyrexia [1]. IL-18 is best known for stimulating NK and T cells to secrete IFN-γ, another broad-activity cytokine. Production of these potent cytokines is tightly regulated: expression requires NF-κB signaling, biological activity requires cleavage of a pro-domain by caspase-1, and secretion is also directed by caspase-1 [1]. Mice unable to signal via IL-1β and IL-18 are more susceptible to several diverse pathogens, including *Shigella*, *Salmonella*, *Candida albicans*, *Staphylococcus aureus*, and influenza virus [1]. However, caspase-1^−/−^ mice are more susceptible to some infections than IL-1β^−/−^IL-18^−/−^ mice [2], underscoring the importance of additional caspase-1 substrates that alter the immune response.

## Pyroptosis

Proinflammatory programmed cell death by pyroptosis is often the terminal fate of a cell with active caspase-1 or caspase-11 [3]. The specific pathways contributing to this complex cellular response are only now becoming defined. Caspase proteolytic activity is required, indicating one or more proteins key to cell survival are cleaved and inactivated. Numerous caspase-1 targets have been identified [4]–[7], but the identity of this/these critical substrate(s) is not yet known. An early step in pyroptosis is formation of small cation-permeable pores in the plasma membrane [8]. This dissipates the cellular ionic gradient and leads to osmotic swelling and lysis [8]. Ca^++^ flux through these pores is responsible for many of the caspase-1–dependent signaling events that will be discussed in this review. Nuclear condensation, DNA fragmentation that is independent of ICAD (inhibitor of caspase(3)-activated DNase), and IL-1β secretion all precede lysis [8]. These features, along with the unique biochemical requirement for caspase-1, distinguish pyroptosis from other cell death programs such as apoptosis, autophagy, necrosis, NETosis, oncosis, pyronecrosis, and necroptosis [9].

Some pathogens take steps to avoid pyroptosis. *Yersinia* sp. avoid inflammation by directly inhibiting pyroptosis [10] and inducing death by non-inflammatory apoptosis [11]. Poxviruses, similarly, inhibit pyroptosis and also suppress IL-18 and IL-1β signaling with receptor antagonists [12]. In contrast, *Salmonella typhimurium*-infected macrophages rapidly activate caspase-1 and undergo pyroptosis. Lysis of infected macrophages releases intracellular *Salmonella* for subsequent phagocytosis and killing by neutrophils [2]. Thus, depriving a replicative niche to intracellular pathogens through pyroptosis is one critical contribution of caspase-1 during some infections.

## Additional Proinflammatory Signals

Damage-associated molecular patterns (DAMPs) such as ATP, DNA, RNA, and heat-shock proteins are strongly proinflammatory when extracellular, and are released during pyroptosis [13]. DAMPs recruit cells to the inflammatory focus, initiate cytokine secretion, and serve as adjuvants for T-cell priming [12]. One DAMP in particular, HMGB1, has a well-understood role in pyroptosis. HMGB1 is a nuclear transcriptional regulator released during pyroptosis that can subsequently be detected by TLR4 and RAGE receptors to stimulate cytokine release and cell migration [14]. In a murine model of endotoxic shock, caspase-1–dependent release of HMGB1 drives inflammation independent of IL-1β, IL-18, or other DAMPs [14].

Members of the eicosanoid family of signaling lipids, such as leukotrienes and prostaglandins, induce vascular leakage and recruitment of cells to the inflammatory focus. Recently, it was shown that the Ca^++^ influx after caspase-1 activation can induce eicosanoid synthesis [15]. Glycine, an inhibitor of cell lysis but not caspase-1–directed secretion pathways [8], did not block eicosanoid release, suggesting release is a secretion event and not due to cell leakage [15]. Caspase-1 activation of this pathway has not yet been characterized in many models of inflammation, but eicosanoid signaling represents a very rapid and severe proinflammatory response initiated by caspase-1.

## Multi-functional Secretory Mechanisms

Unlike typical cytokines that contain signal sequences for canonical ER/Golgi-mediated export, IL-1β and IL-18 reside in the cytosol until caspase-1-dependent secretion mechanisms are activated [1]. IL-1β and IL-18 can be found in secreted plasma membrane, lysosomal, and autophagic vesicles, but appear to often be secreted by other mechanisms that yield soluble, non-vesicle–associated cytokines [16]–[18]. Several other proteins lacking typical secretion signals are released, including a membrane-bound analog of IL-1β, IL-1α, and the growth factor FGF2 [18], induced by the Ca^++^ influx accompanying caspase-1 activation [19]. Ca^++^ influx, either upon cell wounding [20] or pyroptosis [17], also induces exocytosis of lysosomes to help repair membrane lesions [20] and release antimicrobial compounds that can kill extracellular bacteria [17]. Phagocytosed particles and intracellular pathogens are also exocytosed, allowing their removal from the cell prior to lysis [17]. Concurrent release of pathogens, antimicrobial compounds, and DAMPs likely cooperate to amplify the immune response through cross-priming and other mechanisms.

## Remodeling of Cellular Pathways

Several reports have identified links between caspase-1 and autophagy, a program for removal of cellular debris and microbes. These pathways typically have reciprocal activities; active caspase-1 can limit autophagy [21], while autophagy antagonizes caspase-1 activation [22] and depletes both cytosolic IL-1β [23] and inflammasomes [24]. Factors critical for autophagy are involved in numerous cell processes and also function in proinflammatory capacities [25]. Even after caspase-1 activation, autophagy may aid a cell's recovery by limiting further caspase-1 activity and antagonizing lysis [26]. Also promoting cell survival is SREBP, a transcription factor that regulates lipid metabolic pathways involved in membrane repair [27]. SREBP is membrane-bound and inert until proteolytically released; during pyroptosis, caspase-1 cleaves and activates SREBP-regulating proteases [27]. Like autophagy and lysosomal exocytosis, induction of SREBP may help cells recover from low-level caspase-1 activation. These pathways could allow bifurcated responses in which modestly damaged cells survive, while sustained caspase-1 activity in terminally wounded or persistently infected cells can overcome pro-survival pathways.

Proteome-scale analysis of caspase-1 substrates has hinted at additional pathways affected by caspase-1. Substrates of caspase-1 may gain function or have altered localization, but are most likely to lose function from cleavage. Many proteins identified by proteomics, such as cytoskeletal [4], [5], [7], translational [5], [7], and trafficking [5], [7] proteins, may yet prove to be false positives due to their relative abundance and the promiscuity of caspase-1 [6]. One substrate set that has been experimentally verified includes proteins of the glycolysis metabolic pathway: aldolase, triose-phosphate isomerase, glyceraldehyde phosphate dehydrogenase, enolase, and pyruvate kinase [5]. Cleavage of these enzymes disrupts glycolysis, which may contribute to death of the cell, and may also restrict intracellular pathogens by limiting nutrients [5]. Also processed by caspase-1 are other caspases: caspases-4, -5, and -7 [4], [7]. Caspase-4 and -5 are the human homologs of murine caspase-11, which overlaps with caspase-1 function and can be activated in a parallel mechanism [28], [29]. Cleavage and activation of caspase-7 likely accounts for the number of substrates cleaved at caspase-7–like sites during pyroptosis [4], [7]. Caspase-7 is an executioner caspase of apoptosis but is dispensable for pyroptosis [4], indicating death by pyroptosis occurs independently of the apoptosis program.

## Concluding Remarks

Significant work remains to clarify the relationship between PAMP, signaling pathway(s), cell type, and the outputs during pyroptosis. For example, pyroptosis can occur without IL-1β secretion [2], TLR stimulation alone induces monocytes to secrete IL-1β [30], neutrophils secrete comparatively little IL-1β [31], and caspase-1-dependent secretion of eicosanoids is only seen with resident peritoneal macrophages [15]. Additionally, earlier caspase-1^−/−^ mice also lacked caspase-11, and the role of this regulator in pyroptosis merits further investigation [28]. Despite our incomplete understanding of all of these processes, it is clear that integration of inflammatory programs, with caspase-1 as their central regulator, allows a cell to comprehensively react to injury or infection, preventing deleterious delays in initiating immune responses. The robustness of this pathway is normally well-controlled by NLR stringency; however, “hair-trigger” activation of caspase-1 drives inflammation during atherosclerosis, diabetes, Alzheimer's, inflammatory bowel disease, cancer, and several auto-inflammatory genetic disorders. Thus, implications for treatment of the diseases of both industrial and developing countries can come from further study of caspase-1 regulation and signaling.
